# Timing of Spheno-Occipital Synchondrosis Ossification in Children and Adolescents with Cleft Lip and Palate: A Retrospective Case-Control Study

**DOI:** 10.3390/ijerph17238889

**Published:** 2020-11-29

**Authors:** Francisco Vale, Inês Francisco, António Lucas, Ana Roseiro, Francisco Caramelo, Adriana Sobral

**Affiliations:** 1 Faculty of Medicine, Institute of Orthodontics, University of Coimbra, 3000-075 Coimbra, Portugal; ines70.francisco@gmail.com (I.F.); antoniomlucas@gmail.com (A.L.); a-roseiro@hotmail.com (A.R.); adrianasobralmd@gmail.com (A.S.); 2 Faculty of Medicine, Institute of Clinical and Biomedical Research of Coimbra (iCBR), University of Coimbra, 3000-075 Coimbra, Portugal; fcaramelo@fmed.uc.pt

**Keywords:** cleft lip, cleft palate, cone beam computed tomography

## Abstract

Background: Cleft lip and palate (CLP) can affect the development of the maxilla; which may create a midfacial deficiency as well as an interference of the facial growth pattern and dentofacial esthetics. Objective: This study aimed to estimate the chronological age of complete fusion of the spheno-occipital synchondrosis (SOS) in cleft lip and palate patients and a control group; using cone beam computed tomography (CBCT) images. Methods: In this retrospective study; 125 patients were enrolled (cleft lip and palate group (*n* = 91); control group (*n* = 34)). Age comparison was made with a chi-square test; and a Kaplan–Meier analysis determined the median time to reach complete fusion of the spheno-occipital synchondrosis (*p* < 0.05). Results: The experimental group showed statistically significant differences in the median time for complete ossification between males and females (*p* = 0.019). The median time for complete ossification of the spheno-occipital synchondrosis was; for males; 15.0 years in both groups; for females; it was 14.0 years and 13.0 years in the experimental group and in the control group; respectively. Both for males and females; there were no statistically significant differences between experimental and control groups (*p* = 0.104). Conclusions: The present study showed no differences in the ossification of the spheno-occipital synchondrosis between individuals with and without cleft lip and/or palate.

## 1. Introduction

Patients with isolated cleft lip and palate (CLP) have an anatomical defect that may occur during the 4th and 12th weeks of pregnancy [1,2]. CLP prevalence is 1 in 500–2500 live births and has a multifactorial etiology such as geographic location, ancestry, prenatal exposures, maternal age and socioeconomic status [1,2,3].

CLP is known to affect the development of the maxilla by a combination of functional, intrinsic and iatrogenic factors, which may lead to a retruded position of the maxilla and create a midfacial deficiency, negatively influencing the facial growth pattern and dentofacial esthetics [4,5,6,7].

Some studies demonstrate that CLP is not a localized phenomenon and that a deviant morphology can be observed in different structures of the craniofacial complex, such as the basicranium [8,9,10]. There are three important endochondral growth centers in the craniofacial skeleton, namely the sphenoethmoidal synchondrosis, the intersphenoid synchondrosis and the spheno-occipital synchondrosis (SOS) [11]. The SOS is a cartilaginous union between the body of the sphenoid and the basilar part of the occipital bone, and it is the last of the synchondroses of the cranial base to fuse [12,13]. Its growth will influence the anteroposterior dimension of the cranial vault (contributing to 25% of the growth of the skull base) as well as the height and depth of the upper face by moving the anterior cranial base upward and forward and moving its attached maxillary complex away from the foramen magnum [9,11,12,14,15,16]. Previous studies were performed to clarify the relationship between malocclusion and SOS fusion [17,18]. Singh et al. suggested that premature synostosis of the SOS can produce a decrease on the cranial base angle, which may promote a forward movement of the temporomandibular joint, resulting in skeletal Class III malocclusion [17]. These findings were corroborated by Yang et al., who demonstrated that the time and pattern of SOS fusion do not appear to be different in Class I and Class III patients [18].

The SOS fusion is affected by individual growth and maturation, following acceleration and deceleration stages, reaching a plateau at the end of the pubertal cycle [16]. Literature suggests that the average fusion of the SOS normally starts at approximately the age of 7, with a complete ossification between 11 and 14 for females and between 13 and 16 for males, which may be related to early female growth [11,15,19,20,21]. Premature fusion of the SOS has been associated with midface hypoplasia in humans with syndromic craniosynostoses, such as Apert and Crouzon syndromes [19,22,23]. Some studies suggest a delay in maturation or a deviant growth in the early development of the cartilaginous cranial base in CLP patients. However, studies in this field are sparse [9,24].

The purpose of this study was to estimate the chronological age of complete ossification of the spheno-occipital synchondrosis in cleft lip and/or palate patients and a control group, using cone beam computed tomography (CBCT) images. The null hypothesis was that there were no significant differences regarding the chronological age of complete ossification of the spheno-occipital synchondrosis between subjects with and without cleft lip and palate.

## 2. Materials and Methods

### 2.1. Trial Design and Registration

This study was approved by the ethical committee (process number CE-050/2019) of the Faculty of Medicine, University of Coimbra in accordance with the Declaration of Helsinki. All participants signed an informed consent agreement. In this retrospective case-control study, the records were obtained from the database of patients who sought treatment at the Department of Dentistry, between January 2014 and January 2019, which is a convenience sample.

A sample size estimation was carried out using G*Power 3.1.9.2 assuming a bilateral *t*-test, with a ratio of 1:1, a power of 80% and a statistical significance of 0.05. The sample size was computed for the difference of the chronological age of complete ossification between the two groups, which was assumed as relevant if at least one year was found. Considering the standard deviation as two years, the necessary number of subjects in each group was 64.

### 2.2. Selection and Description of Participants

The experimental group consisted of 91 patients with cleft lip and palate, 53 males and 38 females, with a mean age of 11.6 years for males and 12.1 years for females. The patients were classified into four subgroups, namely unilateral CLP (60%), bilateral CLP (23%), isolated labial cleft (9%) and secondary palate cleft (8%).

The control group comprised 34 patients without cleft lip and palate, 17 males and 17 females, with a mean age of 12.3 years for males and 12.7 years for females.

The inclusion criteria for both groups were: (1) age from 7 to 17 years, (2) Caucasian and (3) CBCT scan with a big field of view. The exclusion criteria were (1) syndromes and (2) endocrine and/or metabolic disorders, all according to medical history.

The basic principles of the European Academy of Dental and Maxillofacial Radiology for the use of CBCT were followed in both groups [25]. The CLP patients had medical conditions that required a 3D analysis for the correct diagnosis. Regarding the control group, patients could take CBCT due to several medical conditions, such as impacted teeth, supernumerary teeth and external or internal resorption.

### 2.3. Methods

CBCT scan images were obtained with an i-CAT scanner machine (Imaging Sciences International, Hatfield, PA, USA) set at 0.3 mm voxel size, 120 kV tube voltage, 5 mA current, 100 FOV, 4 s of scanning time and a slice thickness interval of 1 mm, 16 × 10 cm field of view and 0.30 mm^3^ voxel size. The CBCT images were then exported in a format of Digital Imaging and Communications in Medicine and imported into Invivo5 Advanced 3D Imaging Software (Anatomage, San Jose, CA, USA) for image analysis (these images were not manipulated in any way).

**Figure 1:**
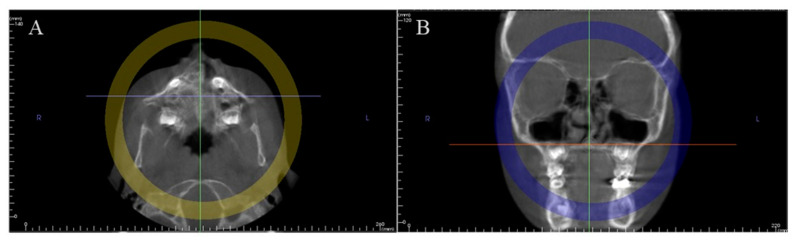
Example of a CBCT record: (**A**) axial view; (**B**) frontal view.

**Figure 2:**
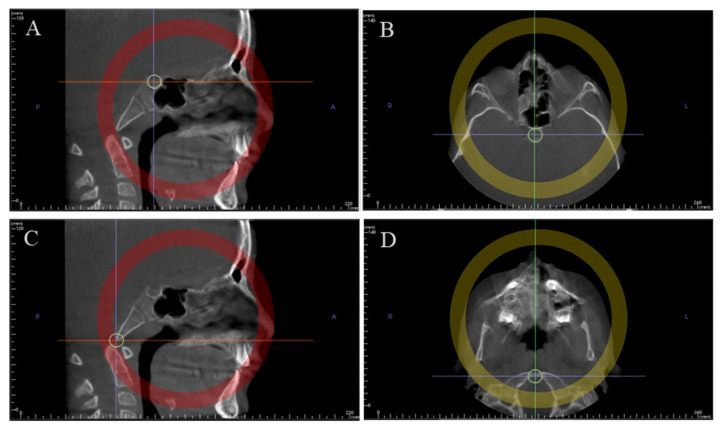
Method used to determine the mid-sagittal plane for evaluation. Middle of sella turcica in the sagittal view (**A**) and in the axial view (**B**); anterior border of the foramen magnum in the sagittal view (**C**) and in the axial view (**D**).

All CBCT images were standardized as follows:

- In the axial view, by positioning the vertical plane in the middle of the anterior border of the foramen magnum (Figure 1A).
- In the frontal view, by leveling the horizontal plane with the palatal plane (Figure 1B).
- A mid-sagittal section of the skull base, passing through the middle of the sella turcica (Figure 2A,B) and the anterior border of the foramen magnum (Figure 2C,D), was considered as the view of choice to assess the SOS. The full extent of the synchondral cartilage was then observed in search of bone bridges. This method increased the total amount of information collected by the 3D exam.

The synchondrosis fusion stage of each patient was assessed by using a five-stage system, proposed by Bassed et al. [26] and modified from that developed by Powell and Brodie [11]. The definition of the staging system is shown in Appendix A and Appendix B. This system was chosen because it is possible to observe a fusion scar on CBCT images (Stage 4), unlike conventional radiographs [26].

### 2.4. Statistical Analysis

Twenty-five random images were selected using Random.org and rescored by the same examiner with a 1-month interval. An intra-examiner agreement was determined using Cohen’s kappa coefficient.

Age distribution was described with a histogram in each group, and each group per sex. A Kolmogorov–Smirnov test was used to assess the differences between age distributions between the two independent groups.

A chi-square test was used to assess the association between sex and group. Although data were acquired within a time period resembling a transversal study, it is important to notice that a time-to-event analysis was performed. The first data point corresponds to the birth moment, for which it was assumed that the event (complete SOS ossification) had not occurred. Considering this rationale, the median time to reach the complete ossification of SOS was evaluated resorting to survival analysis, notably Kaplan–Meier analysis. The definition of the event (complete SOS ossification) was defined as reaching at least stage 4 of the staging system.

All statistical analyses were performed using the Statistical Package for the Social Sciences, version 24.0 for Windows (SPSS Inc., Chicago, IL, USA). A *p*-value of less than 0.05 was considered statistically significant.

## 3. Results

The intra-examiner reproducibility value showed a good agreement with a kappa coefficient of k = 0.920 (*p* < 0.001).

**Figure 3:**
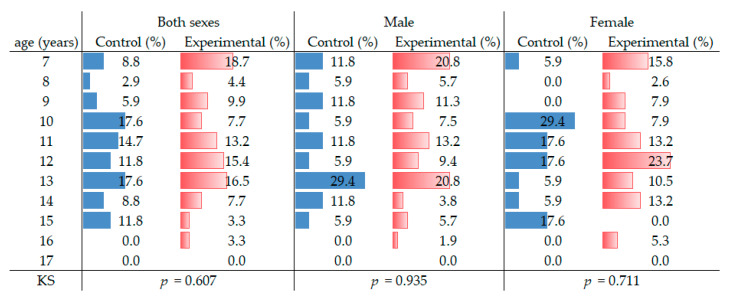
Age distribution histogram for the control and experimental groups.

A total of 125 patients were included in this study and divided into an experimental and a control group. Table 1 and Table 2 show the sample distribution in these groups, respectively. Patients of both groups showed adequate comparability regarding sex (Table 3), and Figure 3 depicts the age distribution by sex in both groups.

**Figure 4:**
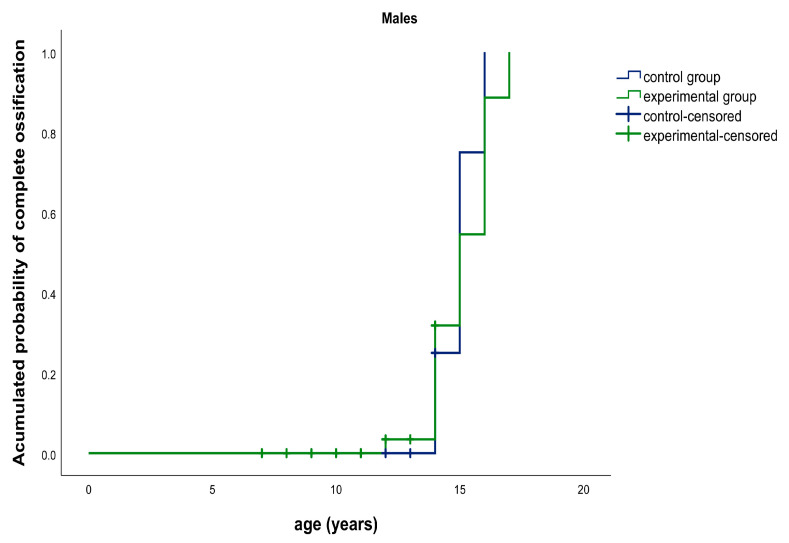
Kaplan–Meier graphic. Median age for complete ossification of the spheno-occipital synchondrosis (SOS): experimental group (green) vs. control group (blue) in males.

**Figure 5:**
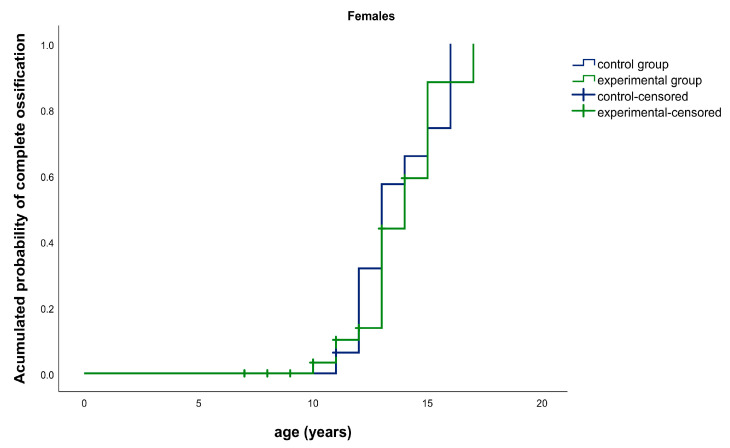
Kaplan–Meier graphic. Median age for complete ossification of the spheno-occipital synchondrosis (SOS): experimental group (green) vs. control group (blue) in females.

In this study, the median time for complete ossification of the SOS was, for males, 15.0 years for both the experimental (CI95% [14.0, 16.0]) and control groups (CI95% [14.2; 15.8]). There were no statistically significant differences between both groups (*p* = 0.806). The results for females were 14.0 years for the experimental group (CI95% [12.7, 15.3]) and 13.0 years for the control group (CI95% [11.9; 14.1]). There were no statistically significant differences between both groups (*p* = 0.565) (Figure 4 and Figure 5).

**Figure 6:**
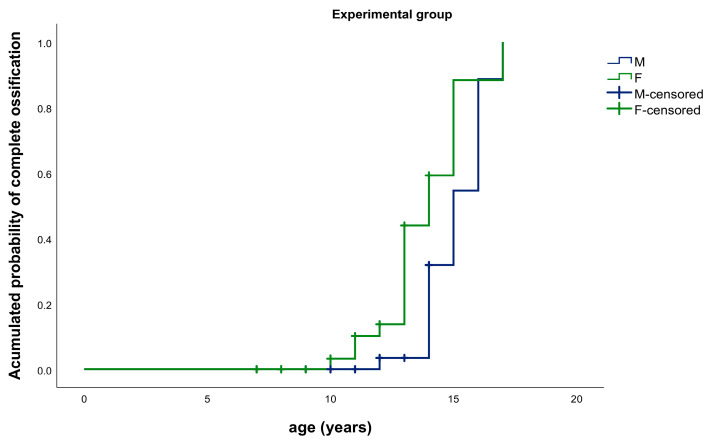
Kaplan–Meier Graphic. Median age for complete ossification of the SOS: experimental group, males (blue) vs. females (green).

In the experimental group, there were statistically significant differences in the complete ossification of the SOS between males and females (*p* = 0.019) (Figure 6).

**Figure 7:**
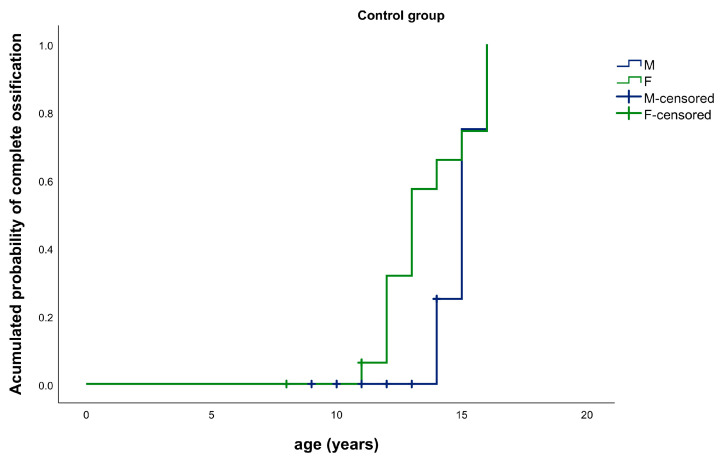
Kaplan–Meier Graphic. Median age for complete ossification of the SOS: control group, males (blue) vs. females (green).

In the control group, there were no statistically significant differences in the complete ossification of the SOS between males and females (*p* = 0.104) (Figure 7).

## 4. Discussion

The purpose of this study was to estimate the chronological age of complete ossification of the spheno-occipital synchondrosis using CBCT images, in cleft lip and/or palate patients and a control group. The endochondral growth centers in the midline of the cranial base have a close structural interrelationship with the nasomaxillary regions during development, influencing the future position of the maxilla [3,5,11,14]. Although this aspect is not well studied in CLP patients, some authors believe that it does not only affect oral and peri-oral structures alone [1,2].

Molsted et al. [27] examined the SOS in lateral cephalograms in newborns with major complete and minor incomplete clefts. They concluded that children with major complete clefts had a broader SOS, which could indicate a delayed maturation or deviant growth in the early development of the cartilaginous cranial base. The same authors, in a different study, also found an increase in the cranial base width and the distance between the left and right ala major of the sphenoid bone in patients with CLP [10]. A lateral skull radiograph can be used to determine the closure of this synchondrosis since it is an inexpensive and conventional exam. While this may be true, it has some disadvantages such as superimposition of structures and low resolution [28]. In recent years, CBCT has become a diagnostic imaging tool in dentistry since it is a three-dimensional imaging modality with lower radiation exposure and with a higher definition, allowing a clearer view of anatomical areas without the superimposition of structures [29,30]. There is a limited number of studies with CBCT regarding cranial base morphology in CLP patients [9,24].

Previous studies reported that the fusion of the SOS occurred two or three years earlier in females than in males [9,11,12,13,14]. However, the timing of complete ossification of the synchondrosis is still controversial in the literature, which is probably due to differences in population, criteria and diagnostic methods [18].

Jahanbin et al. [4] found no significant differences between patients with unilateral CLP, patients with bilateral cleft lip and palate and patients without cleft regarding the middle cranial base length (Ba-S). Liu et al. [9], in a different study, concluded that unilateral CLP patients have smaller Ba-S lengths compared to a normal control group after the end of the pubertal growth peak.

Our study was the first to date to evaluate the chronological age of complete ossification of the SOS in patients with and without CLP. The present study suggests that the median time for complete ossification of the SOS occurs earlier in females (13.0 years for the control group and14.0 years for the experimental group) than in males (15.0 years for both groups), although the statistical significance was not reached in the control group. This finding may be explained by the fact that the control group had fewer individuals and consequently a reduced statistical power, which is a limitation of this study. Nonetheless, these results are in line with the age range described in the literature. The reduced sample in the control group can be justified by following the basic principles of the European Academy of Dental and Maxillofacial Radiology for the use of CBCT, which contributed to the limited number of 3D scans available, according to our inclusion criteria [24,31,32].

Another point of concern, also a limitation of this study, is the uncertainty associated with the occurrence of the event. Since the event is defined by the staging process, one can state that the event has occurred but not know the exact time. The uncertainty of the time of the event leads to a tendency to overestimate the mean and the median time of ossification. The alternative to overcome this aspect would be to design a pure longitudinal study, which would imply the subjects would be submitted to periodic CBCT scans, increasing the accumulated radiation dose. This cumulative radiation is against the “as low as reasonably achievable” (ALARA) principle [33].

Further studies should be conducted to determine the time of complete SOS ossification in patients with CLP, since there is a lack of research in the literature for comparison, probably because CBCT technology has only recently become more common. Additionally, studies with higher sample sizes would be beneficial.

## 5. Conclusions

There are no differences regarding the complete ossification of the spheno-occipital synchondrosis between individuals with and without cleft lip and palate. The complete ossification of this synchondrosis occurs later in males than in females. The use of CBCT scans to evaluate the fusion stage of the SOS could be useful as an additional tool to determine the timing of craniofacial growth and development.

## Figures and Tables

**Table 1:** Sample distribution: experimental group.

Fusion Stage	Sex	Number of Individuals	Mean Age	Min	Max	CI95%	SD
1	M	29	10	7	14	[9.4; 11.1]	2.2
	F	9	9	7	13	[7.3; 10.5]	2.1
2	M	9	11	8	14	[9.4; 12.9]	2.3
	F	3	11	10	12	[8.5; 14.2]	1.2
3	M	3	14	13	14	[12.2; 15.1]	0.6
	F	5	12	8	14	[9.0; 14.6]	2.3
4	M	7	15	12	17	[13.3; 16.4]	1.7
	F	9	12	10	14	[11.3; 13.4]	1.4
5	M	5	15	14	16	[13.5; 15.7]	0.9
	F	12	15	13	17	[13.7; 15.5]	1.4

**Table 2:** Sample distribution: control group.

Fusion Stage	Sex	Number of Individuals	Mean Age	Min	Max	CI95%	SD
1	M	8	10	8	14	[8.5; 12.0]	2.1
	F	3	10	8	11	[5.7; 14.3]	1.7
2	M	4	13	12	14	[11.7; 14.8]	1.0
	F	2	11	11	11	[11.0; 11.0]	0.0
3	M	0	0	0	0	[0.0; 0.0]	0.0
	F	0	0	0	0	[0.0; 0.0]	0.0
4	M	2	14	14	14	[14.0; 14.0]	0.0
	F	8	13	11	16	[11.5; 14.0]	1.5
5	M	3	15	15	16	[13.9; 16.8]	0.6
	F	4	15	14	16	[13.7; 16.8]	1.0

**Table 3:** Intergroup comparisons for sex ratio (chi-square test).

	Experimental Group (*n* = 91)	Control Group (*n* = 34)	
**Sex**			0.409 *
**Male**	53 (58.2%)	17 (50%)	
**Female**	38 (41.8%)	17 (50%)	

* Chi-square.

## Appendix B

### Spheno-Occipital Synchondrosis Fusion Based on a Five-Stage System Proposed by Bassed et al. 2010

**Stage**	**Definition**
1	Synchondrosis is completely open and unfused.
2	Superior border has fused while the remaining fusion site is open.
3	Superior half of the synchondrosis is fused.
4	Complete fusion with a fusion scar in site still visible.
5	Synchondrosis has been completely obliterated with the appearance of normal bone in site.
